# Alteration of Lung and Gut Microbiota in IL-13-Transgenic Mice Simulating Chronic Asthma

**DOI:** 10.4014/jmb.2009.09019

**Published:** 2020-10-08

**Authors:** Kyoung-Hee Sohn, Min-gyung Baek, Sung-Mi Choi, Boram Bae, Ruth Yuldam Kim, Young-Chan Kim, Hye-Young Kim, Hana Yi, Hye-Ryun Kang

**Affiliations:** 1 Institute of Allergy and Clinical Immunology, Seoul National University Medical Research Center, Seoul National University College of Medicine, Seoul 08826, Republic of Korea; 2 Division of Pulmonology, Allergy and Critical Care, Department of Internal Medicine, Kyung Hee University Medical Center, Seoul 0447, Republic of Korea; 3 Department of Public Health Sciences, Graduate School, Korea University, Seoul 02841, Republic of Korea; 4 Laboratory of Mucosal Immunology, Department of Biomedical Sciences, Seoul National University College of Medicine, Seoul 08826, Republic of Korea; 5 School of Biosystems and Biomedical Sciences, Korea University, Seoul 02841, Republic of Korea; 6 Division of Allergy and Clinical Immunology, Department of Internal Medicine, Seoul National University College of Medicine, Seoul 0882, Republic of Korea

**Keywords:** Interleukin-13, asthma, microbiota, gastrointestinal microbiome, microbial interactions

## Abstract

Increasing evidence suggests a potential role of microbial colonization in the inception of chronic airway diseases. However, it is not clear whether the lung and gut microbiome dysbiosis is coincidental or a result of mutual interaction. In this study, we investigated the airway microbiome in interleukin 13 (IL-13)-rich lung environment and related alterations of the gut microbiome. IL-13- overexpressing transgenic (TG) mice presented enhanced eosinophilic inflammatory responses and mucus production, together with airway hyperresponsiveness and subepithelial fibrosis. While bronchoalveolar lavage fluid and cecum samples obtained from 10-week-old IL-13 TG mice and their C57BL/6 wild-type (WT) littermates showed no significant differences in alpha diversity of lung and gut microbiome, they presented altered beta diversity in both lung and gut microbiota in the IL-13 TG mice compared to the WT mice. Lung-specific IL-13 overexpression also altered the composition of the gut as well as the lung microbiome. In particular, IL-13 TG mice showed an increased proportion of Proteobacteria and Cyanobacteria and a decreased amount of *Bacteroidetes* in the lungs, and depletion of Firmicutes and Proteobacteria in the gut. The patterns of polymicrobial interaction within the lung microbiota were different between WT and IL-13 TG mice. For instance, in IL-13 TG mice, lung *Mesorhizobium* significantly affected the alpha diversity of both lung and gut microbiomes. In summary, chronic asthma-like pathologic changes can alter the lung microbiota and affect the gut microbiome. These findings suggest that the lung-gut microbial axis might actually work in asthma.

## Introduction

Asthma is a complex, heterogeneous disease that can be characterized by different cellular, inflammatory, and molecular phenotypes [1]. Decades of research have illustrated a complex and pleiotropic role of interleukin (IL)-13, a key effector cytokine of Th2 inflammation, in allergic disease [2, 3]. For instance, transgenic (TG) mice with lung-specific overexpression of IL-13 presented with hypertrophy of airway epithelial cells, mucus hypersecretion, eosinophilic lung inflammation, and subepithelial fibrosis [4].

Recent advances in the exploration of microbiota by culture-independent sequencing have led to an extended knowledge of the role of commensal microorganisms in asthma. Indeed, microbes play an important role in shaping normal and pathologic immune responses in both lung and gut. For example, increasing evidence suggests a potential role of microbial colonization in the inception of asthma [5]. The composition of the microbiota of asthmatic patients may be modulated by diet [6], antibiotics [7], and infections [8], especially early in life. In addition, recent studies in animal models have focused on the gut microbiota as an important driver of airway allergic inflammation [9-11] and/or oral supplementation of beneficial species (*e.g*., *Bifidobacterium* and *Lactobacillus*) to reduce Th2 cytokine production [12-15]. However, it is difficult to ascertain whether asthma-dependent changes in epithelial structures subsequently induce lung or gut dysbiosis.

In recent years, special focus has been placed upon the microbiota of the airway and gut in the pathogenesis of asthma [16, 17]. This has led to the coining of terms such as the “lung-gut axis.” However, the occurrence of communication between the lung and gut is still under investigation. Despite the emerging study on this lung-gut microbial axis in asthma, it is not clear whether this association is a result of a simple correlation or a causal relationship linked to the pathomechanisms of the disease. If the latter is true, airway microbial dysbiosis might induce alterations in the gut microbiota over time, which in turn would affect other organ immune responses. Several studies have demonstrated that chronic inflammatory airway diseases altered the airway microbiome and had a profound effect on the gut microbiota [12, 14, 18, 19]. However, the causality between emergence of lung diseases and alterations of the gut microbiota is still largely unexplored.

Here, we attempted to characterize the airway microbiome of well-characterized, IL-13-overexpressing TG mice and investigated related secondary alterations in the gut microbiota. Next, we performed an integrative analysis of the lung and gut microbiome of IL-13 TG mice to explore the microbial interactions between lung and gut.

## Materials and Methods

### Lung and Gut Microbiome Specimens from IL-13-Overexpressing TG Mice

The transgenic mice were kindly provided by Professor Jack A. Elias of Brown University [20, 21]. To evaluate the respiratory effector functions of IL-13, we used constitutional IL-13 TG mice with Clara cell 10-kDa protein (CC10) promoter to target the expression of IL-13 in the airway since constitutive IL-13 TG mice can express the transgene without doxycycline, which can affect commensals. Bronchoalveolar lavage (BAL) fluid, lung tissue, and stool were collected for microbiome analysis from 10-week-old, IL-13-overexpressing TG mice (*n* = 30) and their C57BL/6 wild-type (WT) littermates (*n* = 30). Mice were euthanized and two volumes of 1 ml of PBS were infused, gently aspirated, and pooled. Samples were centrifuged at 3,000 ×g for 10 min to recover cells, while the supernatants were collected and stored at -70°C for further analysis. Cell pellets were resuspended in PBS and total cell counts were determined using a hemocytometer. The study protocols were approved by the IACUC of Seoul National University Hospital (Approval No. SNUH-IBC-1606-018-004).

### DNA Extraction and 16S rRNA Gene Sequencing

Genomic DNA was extracted from 1 mL of BAL fluid, two right lobes of lung, and 500 mg of cecum samples using a commercial microbial DNA isolation kit (FastDNA SPIN Kit, MP Biomedicals, USA) according to the manufacturer’s instruction. PCR amplification was performed using the primers 341F (5'- TCGTCGGCA GCGTCAGATGTGTATAAGAGACAGCCTACGGGNGGCWGCAG-3') and 805R (5'-GTCTCGTGGGCT CGGAGATGTGTATAAGAGACAGGACTACHVGGGTATCTAATCC-3') and 2X KAPA HiFi HotStart ReadyMix (KAPA Biosystems, USA). PCR reactions were carried out as follows: 3 min at 95°C, 25 cycles of 30 sec at 95°C, 30 sec at 55°C, 30 sec at 72°C, 5 min at 72°C, and hold at 4°C. A second PCR for the indexing process was performed using the Nextera XT Index Kit (Illumina, USA). The amplicon was purified using a TopQXSEP MagBead matrix (Celemix, Korea) and then sequenced using the MiSeq v3 platform (Illumina).

### Data Analysis

To test differences between the IL-13 TG group and the WT group, the Mann‐Whitney test was used for nonparametric data while an unpaired t-test was used for parametric data. Sequencing data were analyzed using the QIIME pipeline referring to the SILVA 16S rRNA gene database. To evaluate the alpha diversity of bacterial communities, observed operational taxonomic units (OTUs) the Shannon index, the Chao1 index, and the Inverse Simpson’s diversity index were calculated. The beta-diversity of the microbiome was calculated using Principal coordinate analysis (PCoA) and permutational multivariate ANOVA (PERMANOVA) by R package vegan. The differences in taxa abundance were calculated using the Wilcoxon test (*p* < 0.05). Linear discriminant analysis (LDA) effect size (LEfSe) was applied to the OTU table (Kruskal-Wallis test, *p* < 0.05; Wilcoxon test, *p* < 0.05; LDA > 2.0) [22]. Co-occurrence between bacterial OTUs was calculated in terms of Pearson’s correlation coefficients and the results were visualized as a network using Cytoscape. The Benjamini-Hochberg correction for multiple comparisons was applied, and a false discovery rate (FDR) < 0.05 was selected.

## Results

### Lung Inflammation in IL-13-Overexpressing Mice and Preparation of Samples

**Fig. 1:**
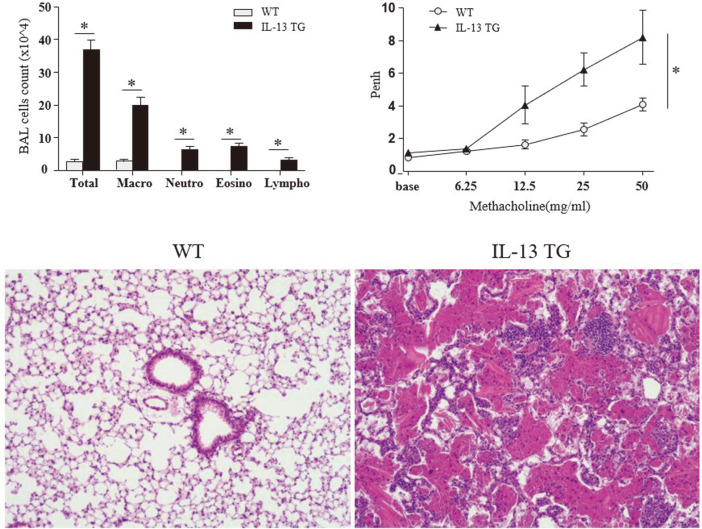
Comparision of total and differential cell count (upper left), airway hyperresponsiveness (upper right), and, histological features of wild-type mice and IL-13-overexpressing transgenic mice. *P-value < 0.05.

To examine the airway inflammatory changes occurring in IL-13 TG mice, we analyzed the extent of airway hyperresponsiveness (AHR) in their lung tissue samples and compared it with that of WT mice. Histopathological analyses revealed that IL-13 TG mice exhibited lung tissue inflammation, in particular eosinophilic infiltration and AHR, which are both typical features of asthma (Fig. 1). Moreover, counting of BAL-derived cells showed a significantly increased total BAL cell count in IL-13 TG mice, associated with an increased number of macrophages, eosinophils, neutrophils, and lymphocytes (*p* < 0.05).

### Assessment of Bacterial Diversity in Lungs and Gut

We compared bacterial diversity of BAL fluids and lung lysates to identify the best method of tissue sampling most accurately reflecting the lower airway microbiota (Suppl. A). However, the amount of bacterial biomass from lung lysates was too low to conduct 16S rRNA gene amplification. Thus, we chose BAL fluids for the current experiment.

**Fig. 2:**
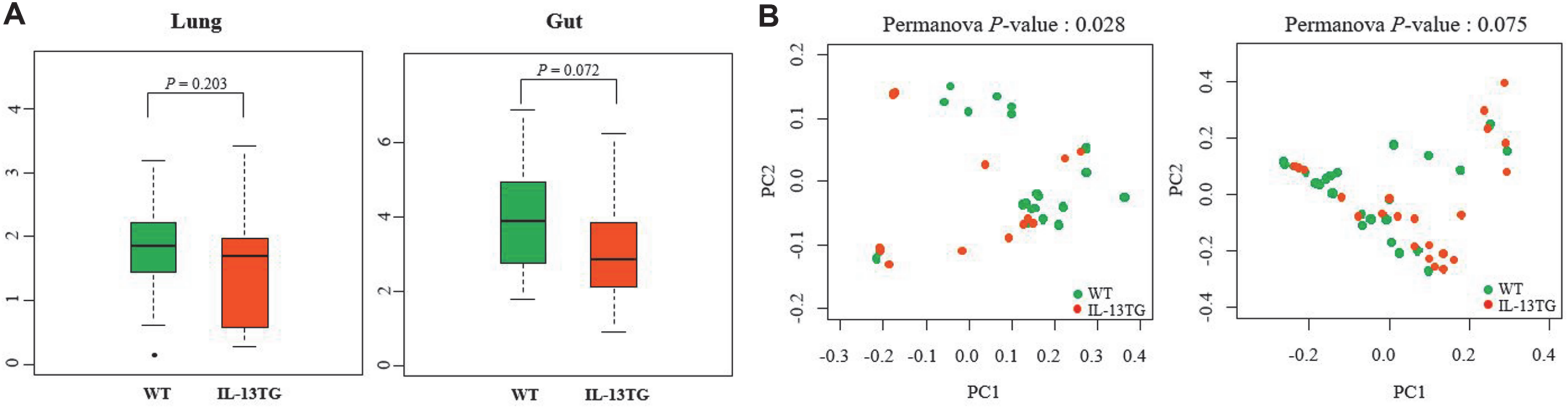
Differences in microbiome diversity between IL-13 TG and WT mice. (**A**) Alpha diversity (Shannon index). (**B**) Beta diversity.

We first compared alpha diversity indicators among groups, which included the number of observed OTUs, the Shannon index, the Chao1 index, and the Inverse Simpson’s diversity index. Microbial communities of BAL samples from the WT mice displayed a higher Shannon index (1.73 ± 0.64) than those from the IL-13 TG mice (1.65 ± 0.90); however, the difference was not significant (*P* = 0.203) (Fig. 2A). Moreover, analysis of alpha diversity of the gut microbiome demonstrated a slight decrease in Shannon index in the IL-13 TG mice with respect to the WT mice, but this difference did not reach statistical significance (WT: 3.98 ± 1.29, TG: 3.26 ± 1.50, *P* = 0.072). However, when plotting matrices of weighted UniFrac distances, the beta diversity of the lung microbiota of IL-13 TG mice was significantly lower than that of WT mice (*P* = 0.028; PERMANOVA) (Fig. 2B). In contrast, the beta diversity of the gut microbiota between the two groups showed a slight, but not significant, difference (*P* = 0.075; PERMANOVA).

### Effect of Lung-Specific IL-13 Overexpression on Lung and Gut Commensals

**Fig. 3:**
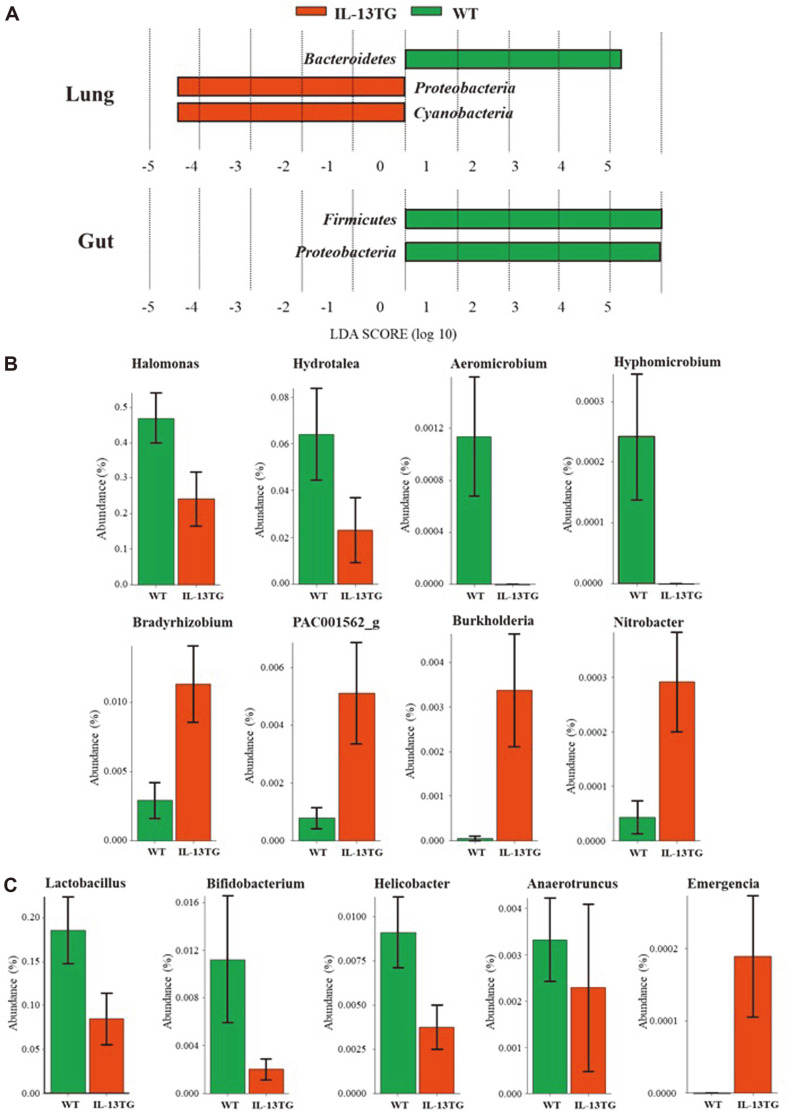
(**A**) Analysis of differences in the composition of the microbiome between IL-13 TG and WT mice by linear discriminant analysis (LDA) effect size (LEfSe). (**B**) Relative abundance (%) of bacterial genera displaying significantly different abundance in the airways of the two groups. (**C**) Relative abundance (%) of bacterial genera displaying significantly different abundance in the gut of the two groups.

We next examined differences in the composition of microbial communities in terms of phyla and genera between IL-13 TG and WT mice. Interestingly, we found significant, highlighted differences in the abundance of certain microbial taxa between the two groups in both lungs and gut (Fig. 3A).

At first, microbiomes were compared at the phylum level. BAL samples of IL-13 TG mice showed reduced abundance of *Bacteroidetes*, and an increased proportion of Proteobacteria and Cyanobacteria compared to those of WT mice. On the other hand, in cecum samples, the abundance of Firmicutes and Proteobacteria observed in WT mice decreased in mice with lung-specific IL-13 overexpression. Interestingly, the relative abundance of Proteobacteria was differentially associated with the lung and gut microbiomes. At the species level, *Halomonas*, *Burkholderia*, *Acinetobacter*, *Klebsiella*, and *Bradyrhizobium* were increased in the lung in the IL-13 TG mice group compared to controls. On the other hand, the proportions of *Helicobacter* and *Desulfovibrio* were decreased in the gut in the IL-13 TG mice, compared to the WT mice.

At the genus level, a total of eight OTUs showed different abundance between BAL samples of WT and IL-13-overexpressing mice (Fig. 3B). In particular, we observed that the abundance of *Halomonas*, *Hydrotalea*, *Aeromicrobium*, and *Hyphomicrobium* decreased in the IL-13 TG group with respect to the WT group. Conversely, the proportion of *Bradyrhizobium*, *Burkholderia*, PAC-001562, and *Nitrobacter* increased in the IL-13 TG group.

In cecum samples, the abundance of four genera, *i.e.*, *Lactobacillus*, *Bifidobacterium*, *Helicobacter*, and *Anaerotruncus*, decreased, whereas only the genus *Emergencia* showed increased abundance in the IL-13 TG group with respect to the WT group (Fig. 3C).

### Polymicrobial Interaction Patterns in Lung and Gut Communities

**Fig. 4:**
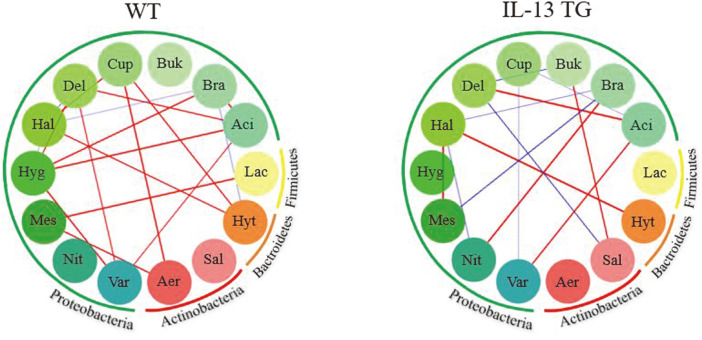
Polymicrobial interaction network between airway microbial communities at the genus level. Red and blue edges represent positive and negative correlations, respectively. (Aer: *Aeromicrobium*, Sal: *Salinispora*, Hyt: *Hydrotalea*, Lac: *Lactobacillus*, Aci: *Acidoborax*, Bra: *Bradyrhizobium*, Bur: *Burkholderia*, Cup: *Cupriavidus*, Del: *Delftia*, Hal: *Halomonas*, Hyg: *Hydrogenophaga*, Mes: *Mesorhizobium*, Nit: *Nitrobacter*, Var: *Variovorax*)

To gain insights into the interaction within lung and gut microbiomes, we performed a bacterial OTU network analysis using CoNet (co-occurrence network interference; http://psbweb05.psb.ugent.be/conet/index.php). Specifically, we calculated Pearson’s correlation coefficients to explore the effect of the IL-13 cytokine on the microbial composition within the BAL and cecum microbiome after filtering by using the top 25 percentile genera (Fig. 4., See Supplement B for all relevant genera data). In the resulting network, each node corresponds to one microbial genus while the color of the edges indicates a positive (red) or negative (blue) relationship. Interestingly, genus-genus interactions within the lung community were considerably different between WT mice and IL-13 TG mice. Indeed, the IL-13 TG mice displayed more competitive interactions presenting negative correlation than WT mice. There was not much interaction within the gut microbiome in either group. Within gut microbial communities, IL-13 TG mice showed a positive correlation between the abundances of Bacteroides and *Lactobacillus*, while in the WT group, the abundances of the genera *Clostridium* and *Adlercreutzia* presented a positive correlation (Suppl. C).

### Microbial Interactions Between the Lung and Gut Communities in IL-13 TG Mice

**Fig. 5:**
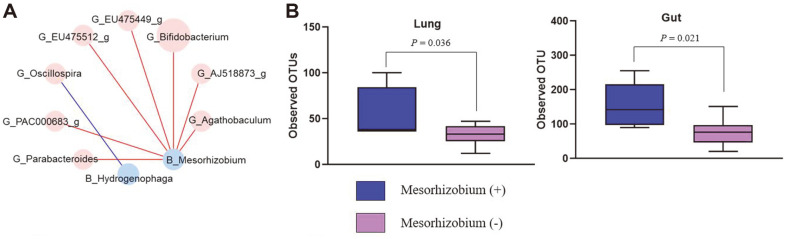
(**A**) Results of multiple comparisons (corrected by the Benjamin-Hochberg method) in lung and gut microbiota of IL-13 TG mice. (**B**) Bacterial diversity based on the presence of *Mesorhizobium* in both lung and gut microbiota of IL-13 TG mice.

Next, we examined the FDR after correction of *p*-values for multiple comparisons using the Benjamini-Hochberg method (Fig. 5A and Supplement D). While there were no obvious relationships between the bacterial composition of BAL and cecum samples in the WT group, two genera in BAL samples and eight genera in stool formed significantly correlated microbial pairs in IL-13 TG mice. Primarily, the genus *Mesorhizobium*, displaying reduced abundance in BAL samples of the IL-13 TG group, was positively associated with *Bifidobacterium*, *Agathobaculum*, and *Parabacteroides* in the gut. We additionally compared microbial diversity based on the presence of *Mesorhizobium* in the IL-13 TG group. Notably, OTU richness in the lung and gut communities was significantly reduced in the absence of *Mesorhizobium* in IL-13 TG (*P* = 0.036 vs. *P* = 0.021, respectively) (Fig. 5B).

## Discussion

In this study, we investigated the effects of IL-13-induced type 2 inflammation on the lung and gut microbiota. Lung-specific IL-13 TG mice showed altered beta diversity and composition of lung microbiome. In addition to lung microbiome, gut microbiome also presented relative deficiency in the phyla Firmicutes and Proteobacteria in IL-13 TG mice. Thus, we herein provide direct evidence that chronic lung inflammation induced by IL-13 can cause dysbiosis not only of the lung microbiota but also of the gut microbiota. Taken together, these findings suggest the potential lung-gut microbiota interaction in a murine asthma model.

The cytokine IL-13 is considered a central effector of allergic asthma which is both necessary and sufficient to induce key features of asthma such as eosinophilic inflammation, mucus production, and airway hyperresponsiveness [23]. In addition, IL-13 leads to subepithelial fibrosis, which contributes to airway remodeling in severe asthma [24]. Comprehensive studies of targeted and inducible pulmonary overexpression of IL-13 in TG mice have defined the role of IL-13 in the cellular and molecular responses to allergic asthma [4, 20, 21]. However, there is scarce evidence on whether an IL-13-rich microenvironment can induce dysbiosis of the lung and/or gut microbiome.

In human, chronic airway inflammation and environmental factors such as diet and antibiotics can shift the lung microbiota towards decreased abundance of beneficial microbiota accompanied by outgrowth of pathogenic microbiota [7,16]. This perturbation in microbial diversity and composition is referred to as lung dysbiosis. Comparing healthy controls with asthmatics, phylum-level abundance has demonstrated dysbiosis characterized by increased Proteobacteria and decreased *Bacteroidetes* in asthmatic airways, which were similar to the findings in this study [25]. We also found eight OTUs showing different abundance in BAL between WT mice and IL-13-TG mice, suggesting future research to evaluate the role of those microbes in the development of lung pathology. Interestingly, a previous study reported that *Halomonas* decreased in the BAL of patients with eosinophilic asthma [26]. Similarly, *Halomonas* reduced in the BAL of IL-13 TG mice. *Burkholderia* is a genus of Proteobacteria and significantly increased in the BAL of IL-13 TG mice compared to WT mice. The proportion of *Burkholderia* in the upper airway microbiome was increased in asthmatics with chronic rhinosinusitis and asthmatics with at least one emergency room visit [27].

A recent study suggested the microbiome interactions are as important as the individual species in shaping fitness traits by mapping gut bacteria against the fruit fly model [28]. Comparison of pairwise correlation between groups showed more negatively correlated microbial pairs in the lungs of IL-13 TG which indirectly implies the competitive condition in the chronic inflammatory microenvironment.

Murine and human studies linked antibiotics use in critical developmental window time to gut dysbiosis and increased asthma risk [29-31]. These data suggest that gut dysbiosis also plays a critical role against chronic airway inflammation by regulating innate and adaptive immune response [32]. We found that the abundance of bacteria producing short-chain fatty acids (SCFAs) upon fiber fermentation, such as *Lactobacillus* and *Bifidobacterium* species, was typically reduced in the gut of IL-13 TG mice, as compared to WT mice. SCFAs, such as acetate, propionate and butyrate, are the most abundant products of anaerobic fermentation of dietary fiber and important metabolites for the maintenance of intestinal homeostasis [33]. Notably, a recent study revealed that *Lactobacillus* supplementation could reduce allergic inflammation and modulate the activity of gut microbes by inducing butyrate biosynthesis in house dust mite (HDM)-sensitized mice [34]. Therefore, the role of SCFAs in the lung-gut microbial interactions remains to be further investigated.

It is well established that disturbances in the gut microbiota during early life can cause allergic asthma, as confirmed by experimental and epidemiological studies [11, 35, 37]. Indeed, an increasing number of studies have revealed that the gut microbiota can influence lung immunity [11, 29]. Thus far, previous studies indicated the primary direction of crosstalk from the gut to the lungs [31,37,38]. On the other hand, whether the lung microbiota affects its gut counterpart is less clear at present. Only one study investigated the influence of acute lung injury on the gut microbiota by lipopolysaccharide instillation [39]. Although the underlying mechanism of microbial communication along the gut-lung axis is not fully understood, several factors are proposed, such as epigenetic modification by bacterial SCFAs [6, 40, 41], enhanced Th 17 inflammation by segmented filamentous bacteria [42], and induction of the NF-κB-dependent pathway via activation of Toll-like receptors by microbes [32]. Our study showed the impact of chronic Th2 lung inflammation on the lung and gut microbiome and should provide new insights into disease pathogenesis and potential therapeutic strategies.

Considering that the lung and gut are structurally similar with identical embryonic origin, these two distant mucosal sites might interact via immune response. Accordingly, previous epidemiologic studies found that individuals with asthma were more likely to develop chronic gastrointestinal comorbidities such as inflammatory bowel disease [43-45]. These findings support the hypothesis that structural and functional alterations in lung epithelium could contribute to gut dysbiosis and vice versa. The lung-gut axis is defined as a shared mucosal immune system along which extensive communication between the lung and the gut takes place, including microbial and immunological changes [17]. Using IL-13 TG mice, we found that chronic lung inflammation induces alterations in the gut microbiota, suggesting a relationship between the lung-gut axis and asthma. However, further investigation is warranted to determine whether gut dysbiosis depends on lung-gut microbial crosstalk or if chronic lung inflammation influences the gut mucosa or the composition of the gut microbiome by inducing inflammation in gut mucosa.

In this study, we found that the lung-gut microbiome interaction showed a different pattern in IL-13 TG mice compared to those of WT mice. To understand the complex and diverse nature of the microbiome, we performed network-based analysis and found that certain genera in airway were associated with abundance of gut microbiota. Particularly, the *Mesorhizobium*, a genus of gram-negative soil bacteria [46], was significantly associated with alpha diversity not only of lung but also of gut in IL-13 TG mice. Although the exact role of *Mesorhizobium* in asthma was not validated in this study, it is interesting to find that there are certain key lung taxa that interact with microbes in the gut and are associated with microbiome abundance in both lung and gut.

Although our data have demonstrated that asthma could alter the microbiome and the polymicrobial interaction between lung and gut, there are still some unsolved issues. First, it is unclear whether IL-13 contributes to gut dysbiosis in a direct or indirect way. IL-13 TG mice simulate chronic asthma which may accompany long standing hypoxia. Further research exploring the effect of IL-13 blockade on lung and/or gut dysbiosis would be helpful to demonstrate the direct effect of IL-13 on the microbiome. Second, this study is limited in ascertaining the directionality of the lung-gut microbial interactions. Further research is needed to determine the direction of lung-gut interactions by transplanting fecal or lung microbiome from IL-13 TG into healthy mice. Third, we did not directly evaluate the SCFAs from cecal samples in IL-13 TG mice for verification of reduction of SCFA-producing bacteria. Finally, we cannot conclude that there is a direct causal-effect relationship between increase in the *Mesorhizobium* and dysbiosis in both lung and gut, although we showed that these genera altered the diversity of both lung and gut in IL-13 TG mice.

In conclusion, our data demonstrated that IL-13-driven type 2 lung inflammation induces not only lung but also gut dysbiosis. Further investigation of microbial communication along the lung-gut axis might provide novel insight into this mechanism in asthma.

## Supplemental Material



Supplementary data for this paper are available on-line only at http://jmb.or.kr.
