# Supplementary material for: Alteration of Lung and Gut Microbiota in IL-13-Transgenic Mice Simulating Chronic Asthma
Source: J Microbiol Biotechnol. 2020 Oct 8;30(12):1819–26. doi: 10.4014/jmb.2009.09019 (PMC9728179; doi:10.4014/jmb.2009.09019)
Supplement: Supplementary file 1 [file JMB-30-12-1819-supple.pdf]

J. Microbiol. Biotechnol.
https://doi.org/10.4014/jmb.2009.09019

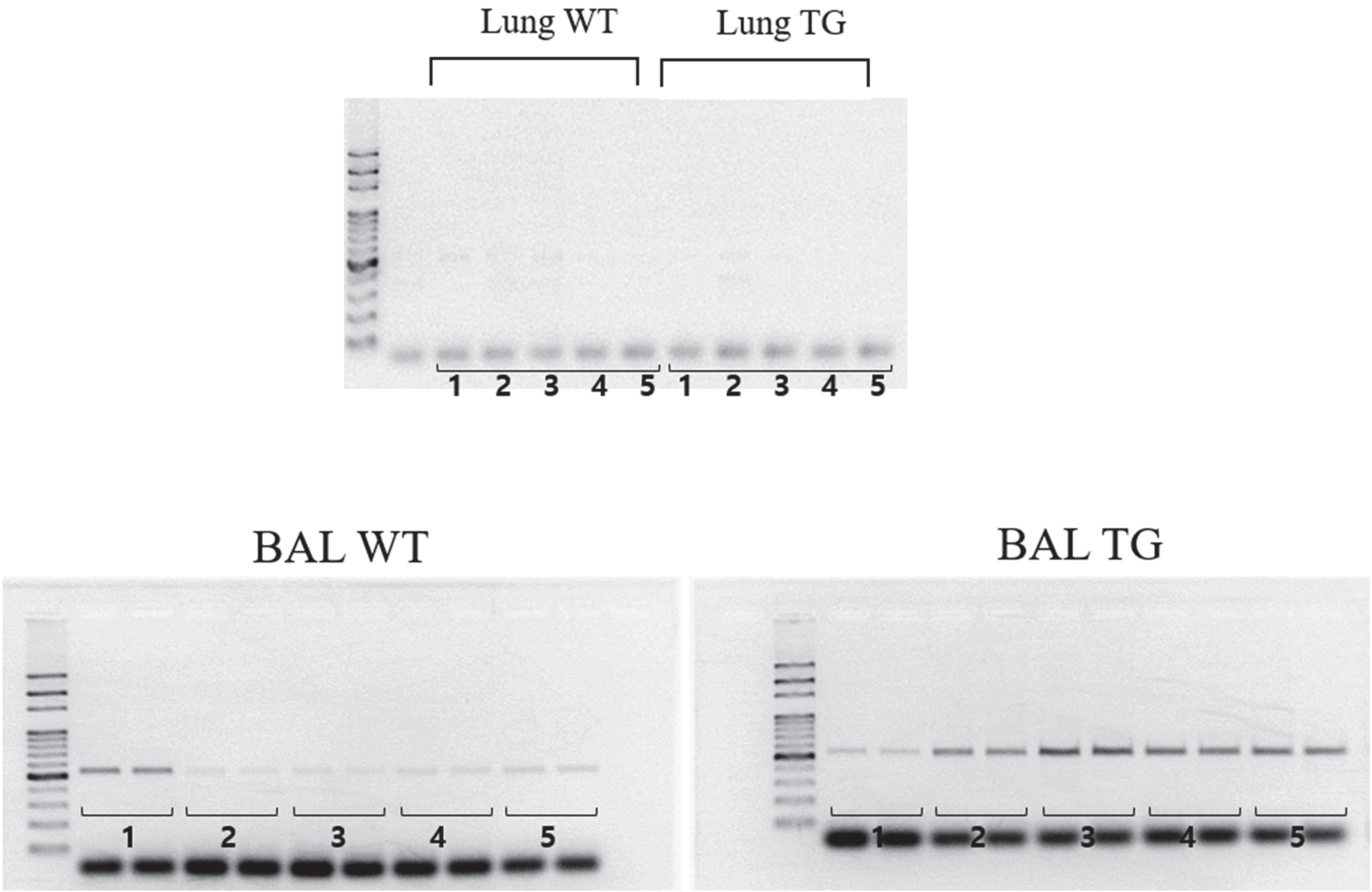

**Supplement B. Polymicrobial interactions within lung microbiome in the wild type mice and the IL-13 TG mice**

| | | **Wild type mice** | | **IL-13 TG mice** | |
|---|---|---|---|---|---|
| | | Correlation coefficient | *P*-value | Correlation coefficient | *P*-value |
| *Halomonas* | *Bradyrhizobium* | -0.742 | <0.001 | -0.793 | 0.001 |
| *Halomonas* | *Hydrotalea* | 0.736 | <0.001 | 0.651 | 0.026 |
| *Acidovorax* | *Variovorax* | 0.979 | <0.001 | 0.976 | <0.001 |
| *Delftia* | *Acidovorax* | 0.741 | <0.001 | 0.674 | 0.019 |
| *Halomonas* | *Mesorhizobium* | - | - | 0.677 | 0.019 |
| *Halomonas* | *Nitrobacter* | - | - | -0.770 | 0.002 |
| *Cupriavidus* | *Acidovorax* | - | - | -0.839 | <0.001 |
| *Cupriavidus* | *Variovorax* | - | - | -0.863 | <0.001 |
| *Bradyrhizobium* | *Mesorhizobium* | - | - | -0.618 | 0.038 |
| *Bradyrhizobium* | *Nitrobacter* | - | - | 0.815 | 0.001 |
| *Delftia* | *Burkholderia* | - | | -0.626 | 0.036 |
| *Delftia* | *Salinispora* | - | | -0.647 | 0.026 |
| *Burkholderia* | *Salinispora* | - | | 0.941 | <0.001 |
| *Halomonas* | *Delftia* | -0.481 | 0.045 | - | - |
| *Halomonas* | *Cupriavidus* | 0.649 | 0.003 | - | - |
| *Halomonas* | *Hydrogenophaga* | -0.604 | 0.008 | - | - |
| *Hydrogenophaga* | *Delftia* | 0.773 | <0.001 | - | - |
| *Hydrogenophaga* | *Acidovorax* | 0.520 | 0.028 | - | - |
| *Acidovorax* | *Bradyrhizobium* | 0.485 | 0.045 | - | - |
| *Hydrotalea* | *Cupriavidus* | 0.715 | <0.001 | - | - |
| *Hydrotalea* | *Bradyrhizobium* | -0.584 | 0.009 | - | - |
| *Variovorax* | *Delftia* | 0.825 | <0.001 | - | - |
| *Variovorax* | *Hydrogenophaga* | 0.583 | 0.009 | - | - |
| *Cupriavidus* | *Aeromicrobium* | 0.496 | 0.040 | - | - |
| *Hydrogenophaga* | *Bradyrhizobium* | 0.587 | 0.009 | - | - |

| | | | | | |
|---|---|---|---|---|---|
| *Mesorhizobium* | *Lactobacillus* | 0.537 | 0.022 | - | - |
| *Mesorhizobium* | *Aeromicrobium* | 0.641 | 0.003 | - | - |

**Supplement C. Polymicrobial interactions of gut genus pairs between IL-13 TG mice and control mice group**

| | | Wild type group | | IL-13 TG group | |
|---|---|---|---|---|---|
| | | Correlation coefficient | *P*-value | Correlation coefficient | *P*-value |
| *Oscillospira* | *Ruminococcus* | 0.717 | 0.007 | 0.744 | 0.021 |
| *Clostridium* | *Adlercreutzia* | 0.651 | 0.029 | - | - |
| *Bacteroides* | *Lactobacillus* | - | - | 0.796 | 0.008 |

**Supplement D. Lung-gut microbial interactions in IL-13 TG mice**

| Lung | Gut | IL-13 TG group | |
|---|---|---|---|
| | | Correlation coefficient | *P*-value |
| *Mesorhizobium* | *Agathobaculum* | 0.826 | 0.007 |
| | *Bifidobacterium* | 0.788 | 0.013 |
| | *Parabacteroides* | 0.752 | 0.025 |
| | *AJ518873* | 0.910 | <0.001 |
| | *EU475449* | 0.802 | 0.013 |
| | *EU475512* | 0.776 | 0.014 |
| | *PAC000683* | 0.789 | 0.013 |
| *Hydrogenophaga* | *Oscillospira* | -0.780 | 0.014 |

`
